# Ecofriendly and low-cost bio adsorbent for efficient removal of methylene blue from aqueous solution

**DOI:** 10.1038/s41598-022-22936-0

**Published:** 2022-11-29

**Authors:** Sabarish Radoor, Jasila Karayil, Aswathy Jayakumar, Jyotishkumar Parameswaranpillai, Jaewoo Lee, Suchart Siengchin

**Affiliations:** 1 Materials and Production Engineering, The Sirindhorn International Thai-German Graduate School of Engineering (TGGS), King Mongkut’s University of Technology North Bangkok, Bangkok, 10800 Thailand; 2 Government Women’s Polytechnic College, Calicut, Kerala India; 3 Department of Science, Alliance University, Bengaluru, Karnataka 562106 India; 4 Department of Polymer-Nano Science and Technology, Jeonbuk National University, 567 Baekje-Daero, Deokjin-Gu, Jeonju-si, 54896 Korea; 5 Department of Bionanotechnology and Bioconvergence Engineering, Jeonbuk National University, 567 Baekje-Daero, Deokjin-Gu, Jeonju-Si, 54896 Korea; 6 Institute of Plant and Wood Chemistry, Technische Universität Dresden, Pienner Str. 19, 01737 Tharandt, Germany

**Keywords:** Environmental sciences, Chemistry, Materials science

## Abstract

A novel bio adsorbent was fabricated from turmeric, polyvinyl alcohol and carboxymethyl cellulose for MB dye removal. The physicochemical, antibacterial and biodegradable nature of the film was evaluated using scanning electron microscopy, optical microscopy, universal testing machine, water contact angle, thermogravimetric analysis, Fourier transform infrared spectroscopy, X-ray diffraction, agar disc diffusion method and soil degradability. The inclusion of turmeric into PVA/CMC film improves the biodegradability, antibacterial activity and thermomechanical property of the films. PVA/CMC/TUR film displayed good MB adsorption capacity (q_e_: 6.27 mg/g) and maximum dye adsorption (R%; 83%) and was achieved at initial dye concentration of 10 mg/L with contact time 170 min at room temperature. The adsorption data of MB on PVA/CMC/TUR film was evaluated using four models Langmuir, Freundlich, Temkin and D-R isotherms. The different kinetic of adsorption (pseudo-first order, pseudo-second order and intraparticle diffusion model) was also applied for adsorption of MB on the films. The experimental result suggests that PVA/CMC/TUR films are an alternate cheap adsorbent for water treatment.

## Introduction

Dyes/colorant are ubiquitous in today’s world and is massively used to give vibrant hue to textiles, paints, food etc.^1–4^. Dyes are also used in cultural festivals for instance Holi in India. In fact, the world without dye is unimaginable. In spite of its wide spread application, dye waste is considered as a menace to the environment^5^. One of the major problems associated with dyes are its nonbiodegradability^6–8^. Dyes dumped from various industries, remains in environment for a longer period of time and destruct the ecosystem^9–12^. Perhaps, water pollution from dyeing industry is one of the severe pollutions faced by the world. Methyl chromium chloride or methylene blue (MB) is one of the oldest synthetic dyes which belong to thiazine family^13^. The vibrant blue colour, high water solubility and low cost make it one of the best choices for colouring fabrics especially wool, silk etc. Methylene blue (MB) is considered as promising drug to treat cyanide poisoning chronic lyme disease, vasoplegic syndrome, encephalopathy etc.^14,15^. In olden times, it was extensively used for the treatment of malaria. Recent studies suggested MB for treating deadly respiratory disease Covid-19. The antioxidant, antidepressant and cardioprotective action of MB is also reported. The other important uses of MB are as staining agent, indicator, photosensitizing agent etc.^16,17^. Contrary MB have many drawbacks, for instance, in large dose (> 2 mg/g) it causes several health issues in humans^18^. Headache, vomiting, dyspnea etc. are some of the mild symptoms of MB poisoning^19^. At high dosage, cardiac arrhythmias, Heinz body formation and pulmonary edema is reported in humans. The high affinity of MB with drug especially psychiatric medicine led to severe damage to the central nervous system^20,21^. The removal of MB from water is necessary to protect the inhabitants and the environment from its harmful effects. Different methods have been employed for the removal of toxic contaminants from water. Filtration, precipitation, flocculation, coagulation, electrochemical reduction, ion-exchange, adsorption, reverse osmosis, membrane filtration, etc. are some of the common methods for the removal of contaminants from water.

Removal of MB from water can be achieved through filtration, precipitation, adsorption, osmosis etc.^22–25^. Adsorption being a simple and cost-effective method is often used for the purification of dye laden water^26–29^. Owing to its effectiveness, several novel adsorbent systems have been fabricated for water remediation process^30^. For instance, cerium phosphate polypyrrole nanocomposite developed using in situ oxidative polymerization removed nearly 96% of Cr (VI) from water. Similarly, polyaniline coated date seed derived biochar was found to be an efficient adsorbent for Cr (VI) remediation^31^. A novel core shell nanocomposite developed from iron oxide and polyacrylamide was reported to have superior MB removal efficiency from aqueous solution^32,33^. Recently, natural materials have been employed for fabricating eco-friendly adsorbents for removing contaminants from water^34–38^. For instance, Pacara Earpod tree (*Enterolobium contortisilquum*) and ironwood (*Caesalpinia leiostachya*) seeds was employed as bio sorbent for the removal of basic fuchsin. Owing to the presence of active functional groups and the porous nature, the bio adsorbent was very effective to adsorb dye^39^. The work led by Reis et al. demonstrated high dye (reactive orange 16 and reactive blue 4) removal efficiency of biobased carbon adsorbent derived from spruce bark residues. The chemical interaction between the dye and the adsorbent is proposed as the main reason for the superior adsorption performance. The adsorption performance was further enhanced by activating the bio adsorbent with ZnCl_2_ and KOH. The maximum adsorption capacity for KOH-BBC1 for RO-16 and RB-4 dyes are 354.8 and 582.5 mg g^−1^ respectively. In another study, the almond shell was employed for dye removal (methyl orange) from aqueous solutions^40,41^.Turmeric/curcumin is one of the extensively explored natural sorbent for water purification. It is obtained from the rhizome curcuma longa which is widely present in southeast Asian countries like India, China etc.^42^. Owing to its bright orange colour and peculiar flavour, it is widely used as spice in Asian food. Perhaps, it is one of the inevitable parts of Indian cuisine. The medicinal property of turmeric is known for many centuries back. The exclusive property of turmeric comes from its active ingredient curcumin. Curcumin is chemically a polyphenol and have plethora of application. The anti-inflammatory, anticancer and antioxidant properties of curcumin is well explored in medicines^43^. Turmeric/curcumin-based adsorbent have received great attention and had been successfully employed for water purification^44^. Kubra et al.^42^ employed turmeric powder for methylene blue removal. The dye uptake was rapid, and 99.5% removal efficiency was achieved even with low adsorbent dosage. Alsheshri et al.^45^ developed a novel eco-friendly adsorbent from curcumin formaldehyde resin for the removal of phenol from aqueous solution. The resin exhibits good adsorption property and 93% of phenol was adsorbed within 60 min. This is attributed to its high surface area and pore size. The adsorbent also shows good antibacterial as well as reusability property. TiO_2_-curcumin nanoparticles is an efficient material to degrade the MB. The high photocatalytic activity of the material promotes the photocatalytic degradation of MB^46^. Inclusion of curcumin in polymer membrane improves its adsorption capacity. For example, polyethersulfone (PES)/curcumin membrane is reported to have good affinity for heavy metal ion such as Ni^2+^, Cu^2+^, Zn^2+^ etc.^47^. Turmeric activated carbon was successfully employed to remove reactive blue MB from water. Recently, zinc curcumin oxide nanoparticle was suggested as a green sorbent for the removal of MB from aqueous medium. The maximum dye adsorption reported was 34.71 mg/g for MB dyes^48^.

Most of the recent report on water remediation involve the use of expensive materials like graphene, carbon nanotube etc. In addition to this, complex methods have been commonly used to fabricate membrane for water purification. Therefore, in recent years there is great demand for the development of eco-friendly and biodegradable materials for water treatment. Hence, in this article we report the development of a novel film using eco-friendly and naturally occurring materials such as turmeric, CMC and PVA. Polyvinyl alcohol (PVA) and carboxymethyl cellulose (CMC) was selected as the base polymers. Both these polymers are eco-friendly and have received great attention for water purification applications^49,50^. The film was synthesised using a cost-effective and simple technique; solvent casting. This is the first report on the development of PVA/CMC/TUR film for MB removal from aqueous solution. The crystallinity, chemical structure, thermal, mechanical, wettability and antibacterial property of films was evaluated through XRD, FTIR, thermogravimetric, UTM, contact angle and disc diffusion method. The adsorption properties were thoroughly studied by varying the initial dye concentration, turmeric dosage, contact time, temperature and pH. The adsorption isotherm was investigated using Langmuir, Freundlich, Temkin and D-R isotherms models. The kinetics of adsorption was evaluated using pseudo-first order (PFO), pseudo-second order (PSO) and intraparticle diffusion model (IPD). Thermodynamics of the process was also tested at three different temperatures i.e., 303, 313 and 323 K. The antibacterial activity and biodegradation ability of the film was evaluated. Finally, adsorption–desorption studies of the film were also reported.

## Materials and methods

### Reagents

Polyvinyl alcohol (PVA, (C_2_H_4_O) was obtained from Ajax Finechem Pvt. Ltd. Carboxymethyl cellulose (CMC, (C_6_H_7_O_2_(OH)_x_(OCH_2_COONa)_y_)_n_) was procured from Chemipan (Thailand). The cross-linking agent glutaraldehyde (C_5_H_8_O_2_; GA) and methylene blue (MB, C_16_H_18_ClN_3_S) was purchased from Loba Chemie Products Limited. (Thailand). Turmeric powder was supplied from Bangkok, Thailand. The chemical materials were of analytical grade and were used without further purification. The chemical structure, molecular weight and λmax of MB dye is shown in Table 1.

**Table 1:** The chemical structure of methylene blue.

Name	MW (g/mol)	λmax (nm)	Structure
Methylene blue (MB)	319.85	664	

### Fabrication of polyvinyl alcohol/carboxymethyl cellulose/turmeric film

**Figure 1:**
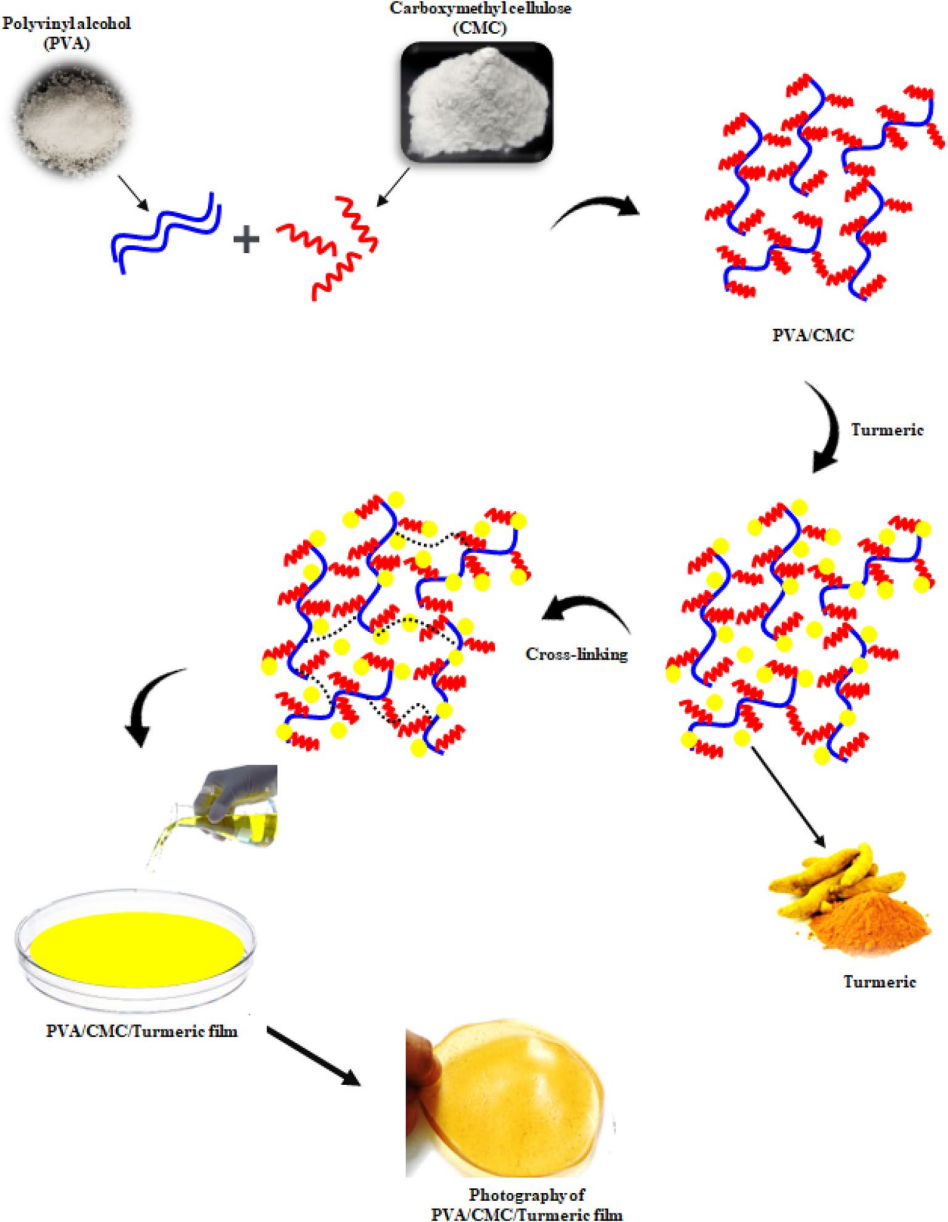
Schematic representation of PVA/CMC/TUR film casting.

**Figure 2:**
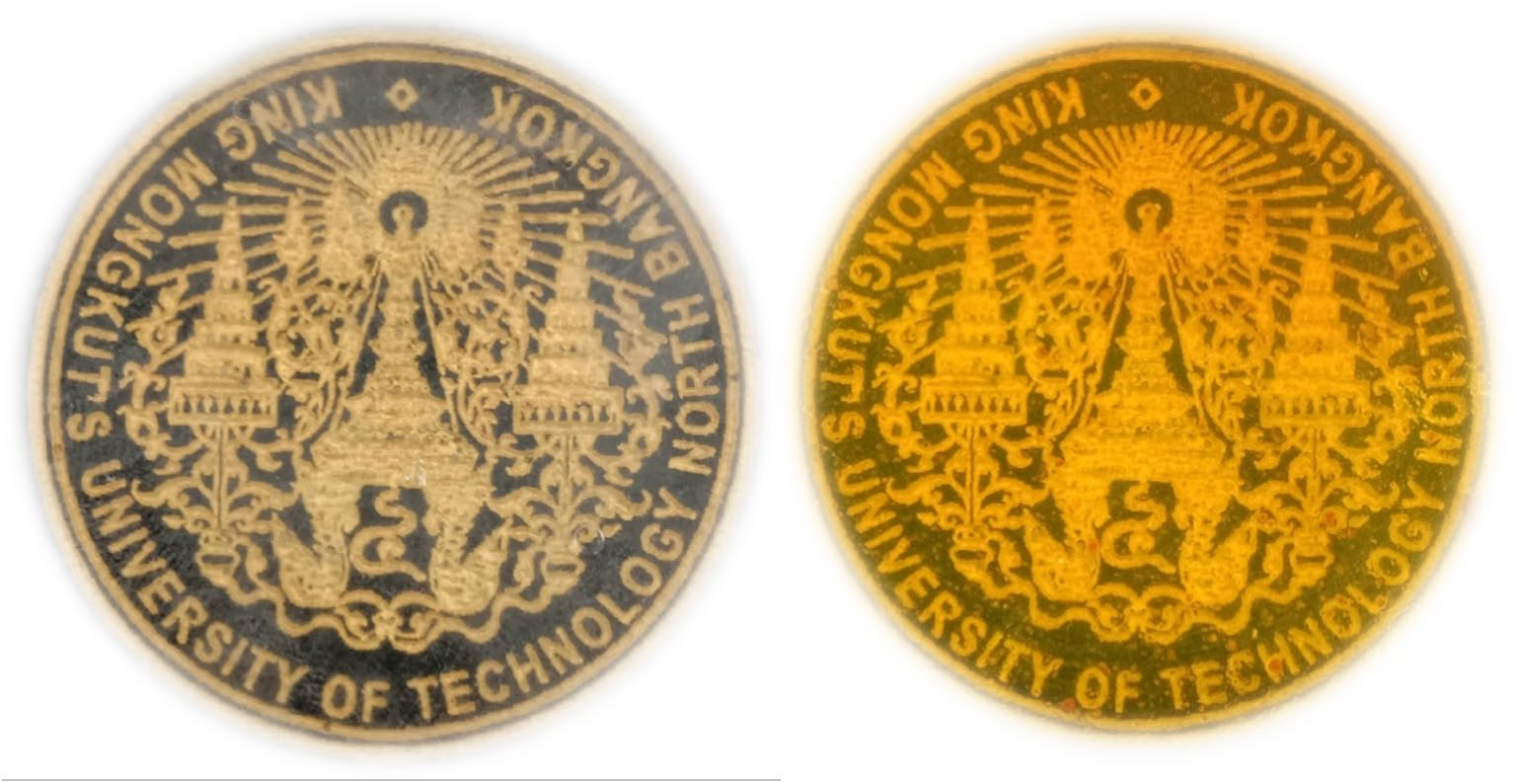
Photographs of plain PVA/CMC and PVA/CMC/TUR films.

Polymer solutions (PVA and CMC) was prepared separately by dissolving the required amount of polymer in distilled water. Later, the two solutions i.e., PVA and CMC were mixed with constant stirring at room temperature to get a clear homogenous solution. This was followed by the addition of different weight percentage of turmeric powder. The composition of the films is illustrated in Table 2. Glutaraldehyde is an effective and economical cross-linking agent for amine, hydroxy and thiol functional group. Hence, we used GA in presence of acid catalyst (HCl) to crosslink PVA and CMC. The crosslinked polymer solution was then poured into clean petri dish and kept for drying at 40 °C for 12 h. The polymer film loaded with 0, 1, 2 and 3wt.% of turmeric powder was labelled as PCT-0, PCT-1, PCT-2 and PCT -3 respectively. The schematic representation of solvent casting method is shown in Fig. 1. The synthesized film possesses good transparency, and the photographs of the film are provided in Fig. 2. The film thickness was measured using screw gauge at different position and a uniform thickness of (27 ± 1.0 µm) was noted.

**Table 2:** The composition of the PVA/CMC/TUR film.

Sample code	PVA (wt%)	CMC (wt%)	Turmeric (wt%)
PCT-0	10	1	0
PCT-1	10	1	1
PCT-2	10	1	2
PCT-3	10	1	3

### Characterization

#### X-ray diffraction

The X-ray diffraction pattern of the PVA/CMC/TUR film was obtained using Riguku SmartLab X-ray diffractometer with CuKα monochromatic radiation. The samples were scanned from 5 to 90° with a speed of 2°/min.

#### Fourier transform infrared spectroscopy (FT-IR)

The chemical composition of the films was examined using FTIR spectrometer (Invenio S, Bruker). The FTIR spectrum of the film was recorded from 400 to 4000 cm^-1^ in transmission mode with a resolution of 4 cm^-1^.

#### Scanning electron microscopy (SEM)

The morphological analysis of the film was evaluated using scanning electron microscope (FEI Quanta 450) with accelerating voltage of 5 kV.

#### Optical microscopy

The surface morphology of the film was also analysed using optical microscopic (Olympus BX43 series).

#### Universal testing machine (UTM)

The mechanical test of the films was performed using universal testing machine (Cometech QC-508B2) with a 500 N loaded cell with crosshead speed of 5 mm/min. Films was cut into 60 mm (length) × 10 mm (width) dimension. Each experiment was conducted at least five times and the average values were taken. The experiments were performed using ASTM standard.

#### Thermal analysis

The thermal behaviour of the films was identified by thermogravimetric analysis (Mettler Toledo TGA/DSC 3 + HT/1600). The temperature range chosen for the current study is from 50 to 750 °C at a heating rate of 10 °C/min under nitrogen atmosphere.

#### UV–vis spectroscopy (UV–vis)

The solid and liquid adsorption studies of the film were evaluated with a UV–VIS spectrophotometer (UV-210 Specord) at wavelengths ranging from 200 to 800 nm.

#### Water contact angle

Contact angle measurement of the film was measured using Drop Meter SCA data physics. The test was performed by placing 2 µL of water droplet on the surface of the film at 25 °C. The image of the water droplets was captured using a digital camera. The angle between the baseline of water droplet and the surface of the film was recorded as the contact angle. All the experiments were repeated five times at different position of the film and the results were averaged.

#### Antibacterial properties

The antibacterial activity of PVA/CMC/TUR films was tested against gram-negative bacteria (*E. coli*) and gram-positive bacteria (*S. aureus*) by inhibition zone method. Initially, the films were punched into circular shape and later placed on Mueller Hinton petri plate inoculated with the gram-positive (*S. aureus*) and gram-negative (*E. coli*) bacteria. The agar plate was kept for incubation at 37 °C for 24 h. After the incubation period, the inhibition zone formed around the samples was measured.

#### Degree of swelling

Water swelling experiment was performed to determine the durability of the films in aqueous medium. For the study, a pre-weighed film of dimension 2 × 2 cm was soaked in 20 mL distilled water for 24 h at 25 °C. The swollen film was removed from immersion, wiped with filter paper and the weight gain was recorded. The process was continued until a constant weight is obtained. The swelling degree of the film was calculated using the Eq. (1)

1 $${\text{Swelling ratio }} = \frac{{{\text{W}}_{{\text{s}}} - {\text{ W}}_{{\text{d}}} }}{{{\text{W}}_{{\text{d}}} }} \times 100$$

W_s_ and W_d_ represents weight of the swollen and dry films respectively.

#### Soil degradation test

The biodegradability of PVA/CMC/TUR film was conducted by exposing the sample to soil at room temperature. Initially, a 2 × 2 cm sample is cut from the film and its initial weight is recorded. The sample is then placed in the agricultural mud for 120 days at a depth of 6 cm and the humidity of the soil was maintained by sprinkling the water on alternate days. After 120 days, the sample was taken out, washed with water, dried at 70 °C in an oven and the weight loss was noted^51,52^.

### Adsorption experiment

Batch experiment was performed to investigate the influence of various parameters such as initial dye concentration, pH, temperature, turmeric dosage and contact time on the adsorption process. Adsorption studies was conducted by immersing 0.04 g of PVA/CMC/TUR film in 30 mL of MB solution of different concentration (10–50 mg L^-1^). The dye solution was drawn at different time intervals, and concentration of absorbed MB dye was monitored using UV–Visible spectrophotometer (Specord (UV-210)) at λ_max_ = 664 nm. Finally, the removal percentage (R%) and equilibrium adsorption capacity q_e_ (mg/g) of MB dye was calculated using the equations^53^;

2 $${\mathrm{q}}_{\mathrm{e}}=\frac{({\mathrm{C}}_{0}-{\mathrm{C}}_{\mathrm{e}})\mathrm{V}}{\mathrm{m}}$$

3 $$\mathrm{R}(\mathrm{\%})=\frac{{\mathrm{C}}_{0}-{\mathrm{C}}_{\mathrm{e}}}{{\mathrm{C}}_{0}}\times 100$$

where C_0_ and C_e_ (mg/L) are the concentrations of MB solution before and after adsorption, respectively; V (L) is the volume of the aqueous MB solution and W is the weight of the film (g). The adsorption studies were monitored by changing the pH (2–10) and the temperature (30–50 °C) of the solution.

### Desorption experiment

The desorption of MB from PVA/CMC/TUR film was evaluated by immersing the film into 30 mL aqueous MB (10 mg L^-1^) solution. After saturation with dye, the film was taken out and mixed with 0.1 N HCl solution for 2 h. Later the film was removed from the solution, washed with distilled water, dried and reuse it. The reusability experiment was monitored at least 5 times using UV–Visible spectrophotometer^54^.

## Result and discussions

### Characterisation of polyvinyl alcohol/carboxymethyl cellulose/turmeric film

**Figure 3:**
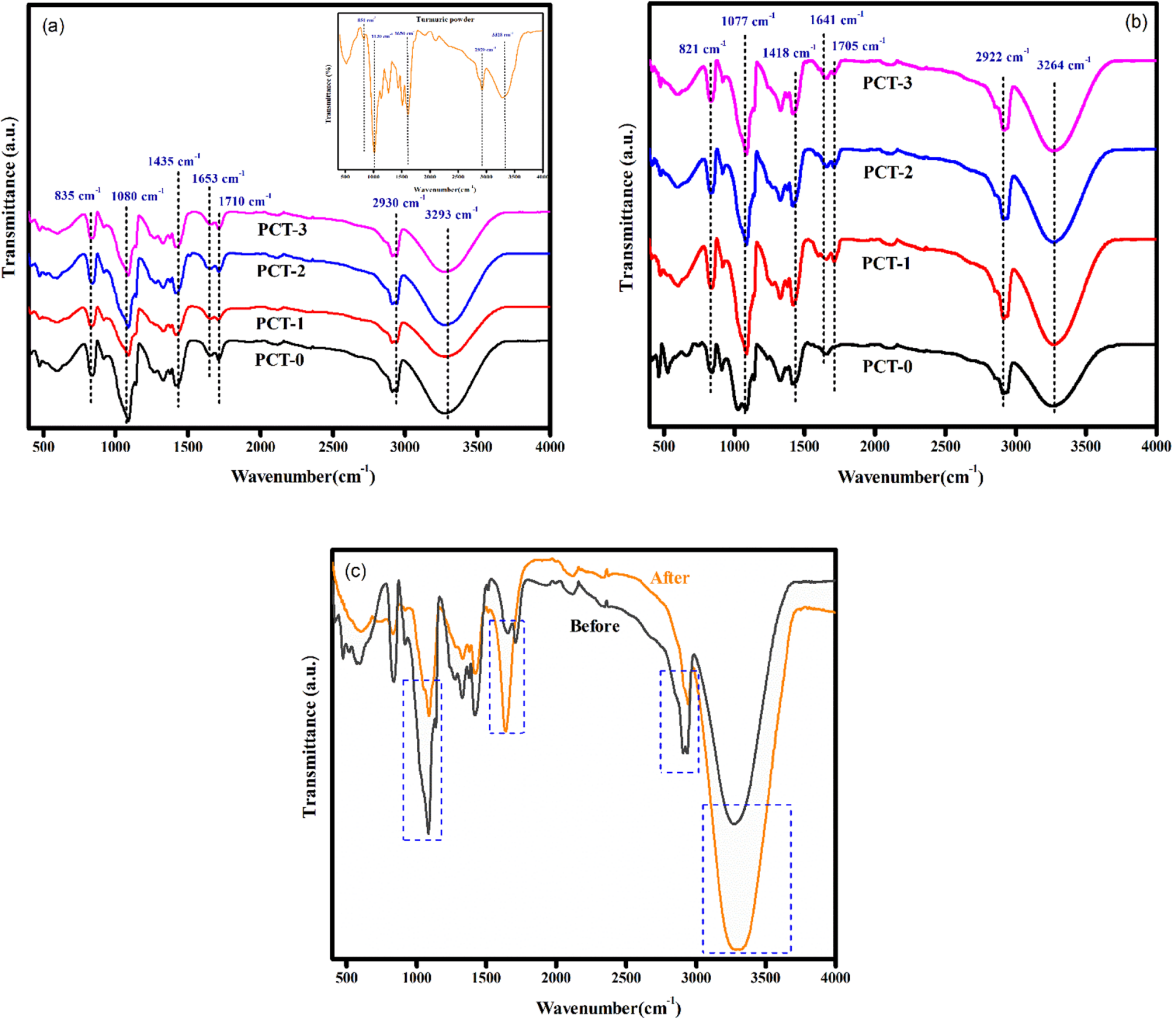
FTIR spectra of: (**a**) PVA/CMC with different percentage of turmeric (inset: turmeric powder) (**b**) MB dye on PVA/CMC/TUR film (**c**) Enhancement of peak intensity after MB adsorption on PVA/CMC/TUR films.

**Figure 4:**
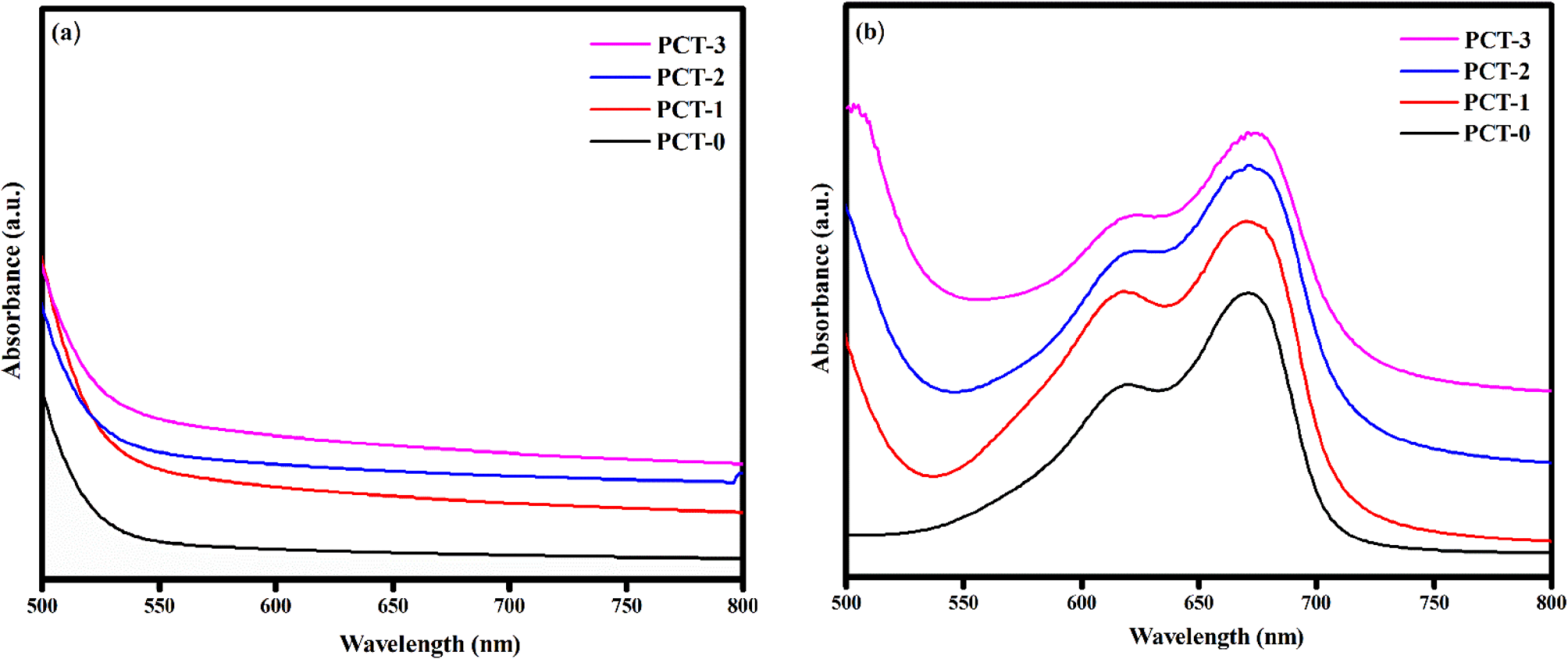
UV–Vis spectra of PVA/CMC/TUR films: (**a**) before and (**b**) after adsorption of MB.

**Figure 5:**
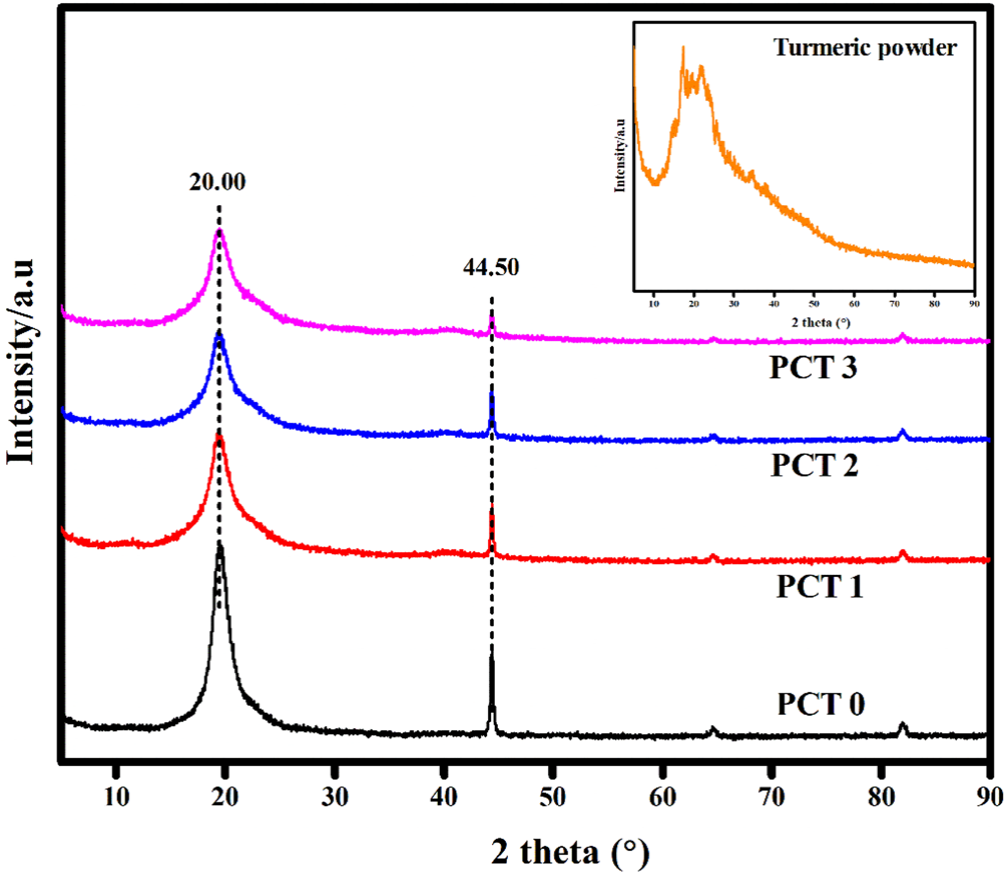
X-ray diffraction (XRD) patterns of turmeric incorporated PVA/CMC films (inset: turmeric powder).

The structural features and chemical interaction are elucidated using FTIR spectroscopy. Turmeric powder displayed characteristic peak at 3328 cm^-1^ (–OH stretching), 2920 cm^-1^ (C–H stretching), 1650 cm^-1^ (C=O stretching), 1130 cm^-1^ (C–O stretching) and 851 cm^-1^ (C–O–C stretching). These characteristic peaks are clearly seen in the FTIR spectra of PVA/CMC/TUR film^55,56^. It can be noted that the hydroxyl peak of the film showed a blue shift of approximately 35 cm^-1^. Also, the broadness of hydroxyl peak decrease with turmeric dosage. The result thus confirmed the presence of intermolecular hydrogen bond between turmeric and the polymer. The FTIR spectra of PVA/CMC/TUR before and after MB adsorption was also recorded to understand the interaction between the dye molecule and the film (Fig. 3a,b). It can be clearly seen that after dye adsorption, the characteristic peak of the films observed at 3269, 2922, 1643, 1077 and 821 cm^-1^ is shifted, and the enhancement of the peak intensity is noted (Fig. 3c). This indicates strong electrostatic interaction between the positive charged MB molecule on the surface of negative charged film^57,58^. The FTIR analysis was further strengthened by the UV analysis. The UV spectra of the film before and after MB adsorption is presented in Fig. 4a,b. It is quite evident from the figure that after dye adsorption, a new band at 664 nm was noted. This peak is the characteristic of MB dye molecule and thus confirmed the adsorption of MB on the film. The X-ray diffractogram of neat PVA/CMC and turmeric incorporated PVA/CMC films is shown in Fig. 5. The films displayed sharp peaks at 2θ = 20° and 44.50°, corresponding to the semicrystalline nature of PVA and CMC. The intensity of this crystalline peak is slightly diminished after the addition of turmeric, probably due to strong interaction between the turmeric and the polymers^59,60^.

**Figure 6:**
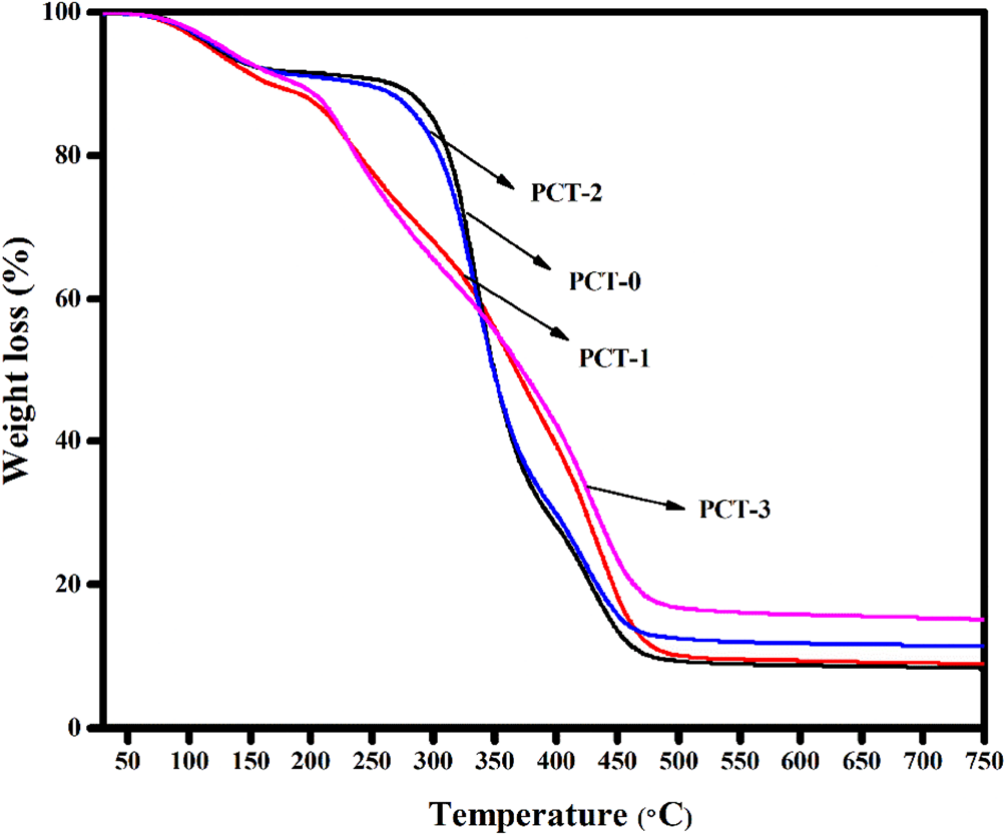
Thermogravimetric (TGA) curves of PVA/CMC/TUR films.

**Figure 7:**
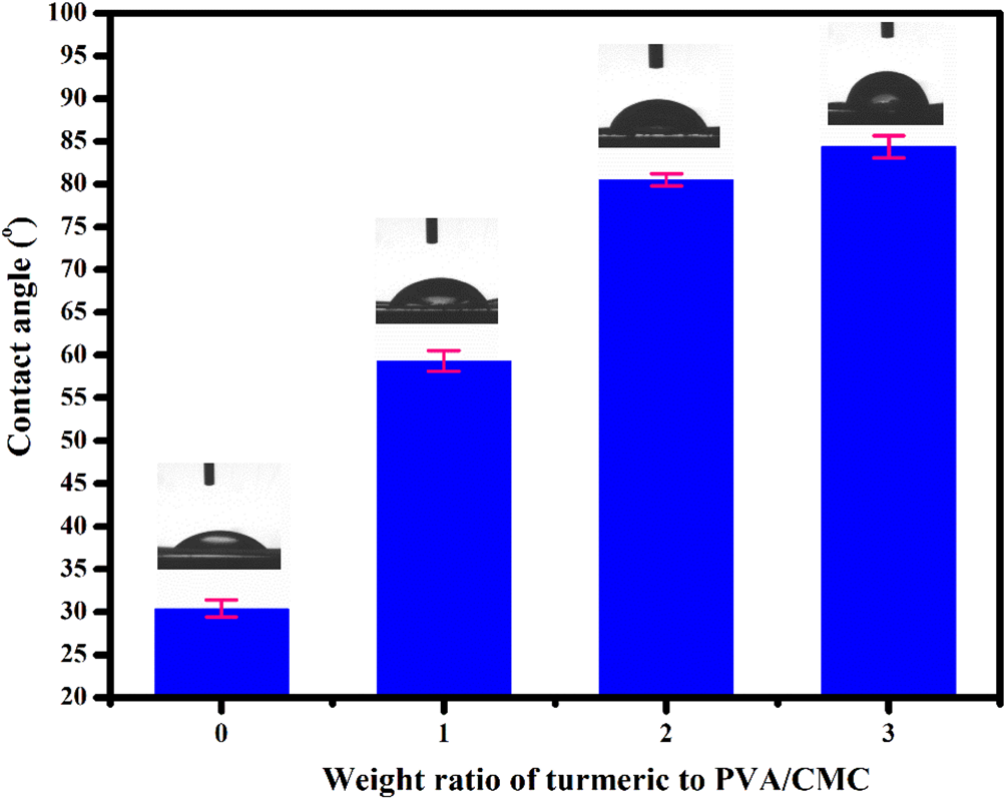
Water contact angle of PVA/CMC/TUR films.

Figure 6 show the TGA curves of neat PVA/CMC and turmeric incorporated PVA/CMC films. The weight loss stages observed at 30–180 °C and 180–400 °C is attributed to the decomposition of physical absorbed water molecules and structural degradation of CMC respectively^61^. The third weight loss in the range of 400–650 °C corresponds to the decomposition of PVA and turmeric. It can be also seen that the film loaded with high weight percentage of turmeric (PCT-3) has high decomposition temperature. Table 3 shows the weight loss of the films at different temperature. It can be clearly seen that on increasing the turmeric content from 1 to 3 wt%, the total weight loss of the film decreases from 90.78 to 84.16%. Hence, turmeric loaded film is thermally more stable than neat films^62^. The surface wettability of film was evaluated by measuring the water contact angle (Fig. 7). A low contact angle (> 90°) indicate a hydrophilic surface. Meanwhile, contact angle greater than 90° represents a hydrophobic surface*.* The water contact value for PVA/CMC/TUR film (PCT-1, PCT-2, PCT-3) are 59.3°, 80.5° and 84.4° respectively. As the content of turmeric increases, a 3% increase in the value of contact angle was noted. This is not surprising as the turmeric is more hydrophobic than PVA and CMC^63^.

**Table 3:** Weight loss at different temperature on PVA/CMC/TUR films.

Samples code	Weight loss (%)			Total weight loss (%)
	30–180	180–400	400–650	
PCT-0	8.22	64.32	18.43	90.97
PCT-1	11.02	49.49	30.27	90.78
PCT-2	7.62	63.11	16.03	86.76
PCT-3	8.62	48.89	26.65	84.16

**Figure 8:**
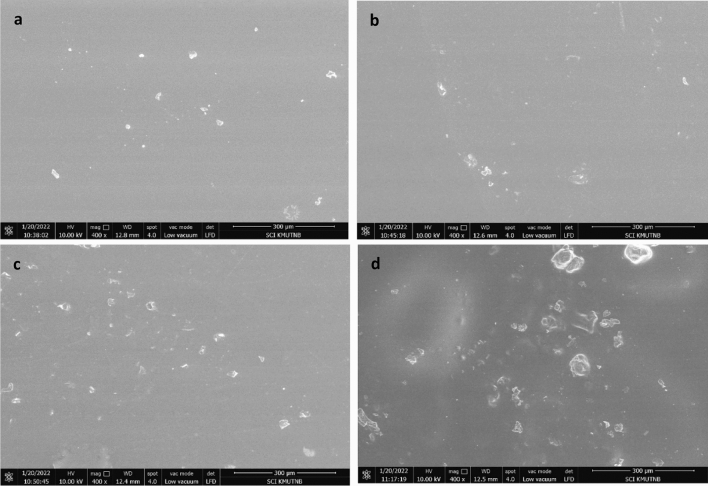
SEM images of PVA/CMC/TUR films: (**a**) PCT-0 (**b**) PCT-1 (**c**) PCT-2 (**d**) PCT-3.

**Figure 9:**
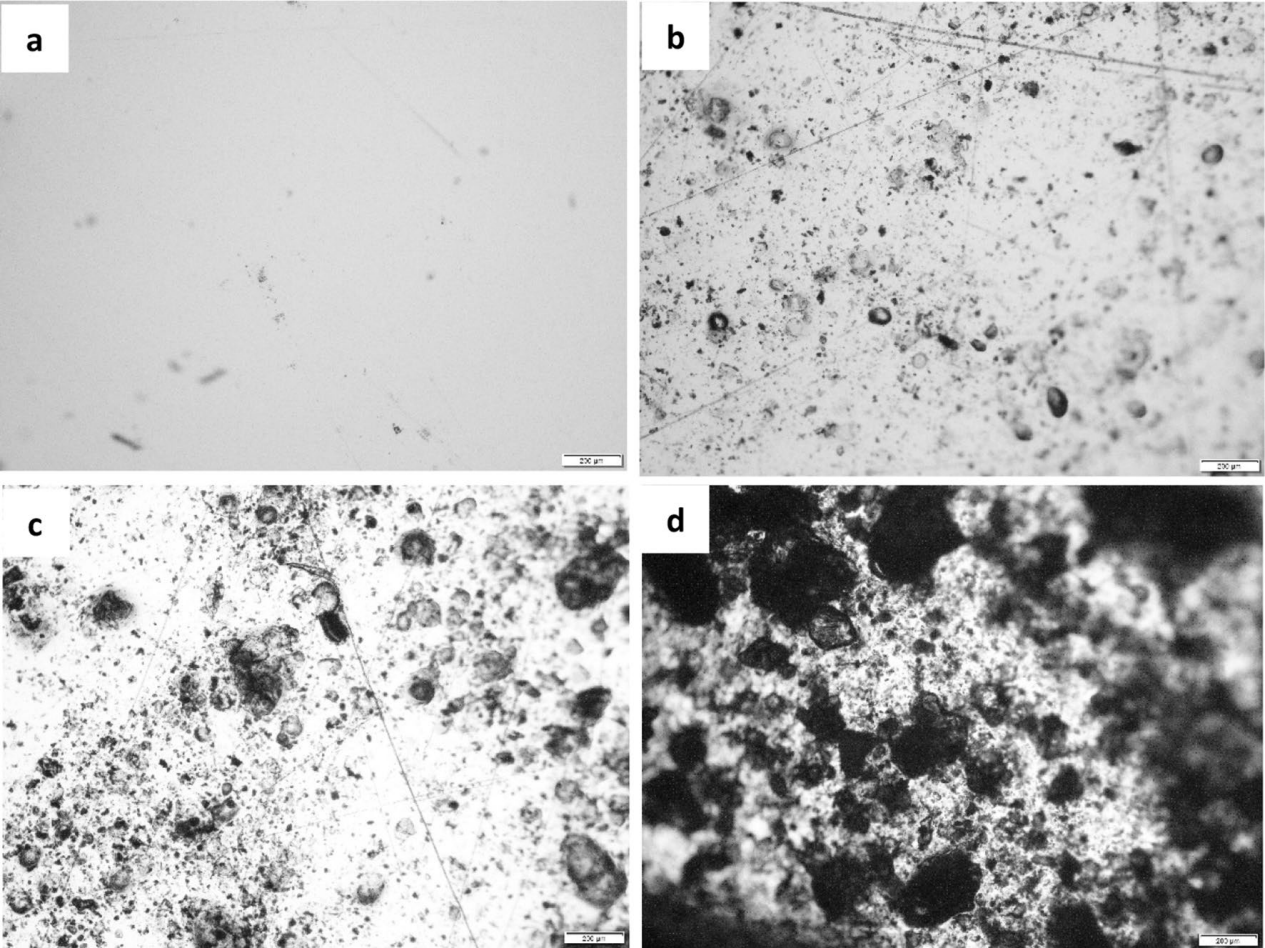
Optical microscopic image of PVA/CMC/TUR films: (**a**) PCT-0 (**b**) PCT-1 (**c**) PCT-2 (**d**) PCT-3.

**Figure 10:**
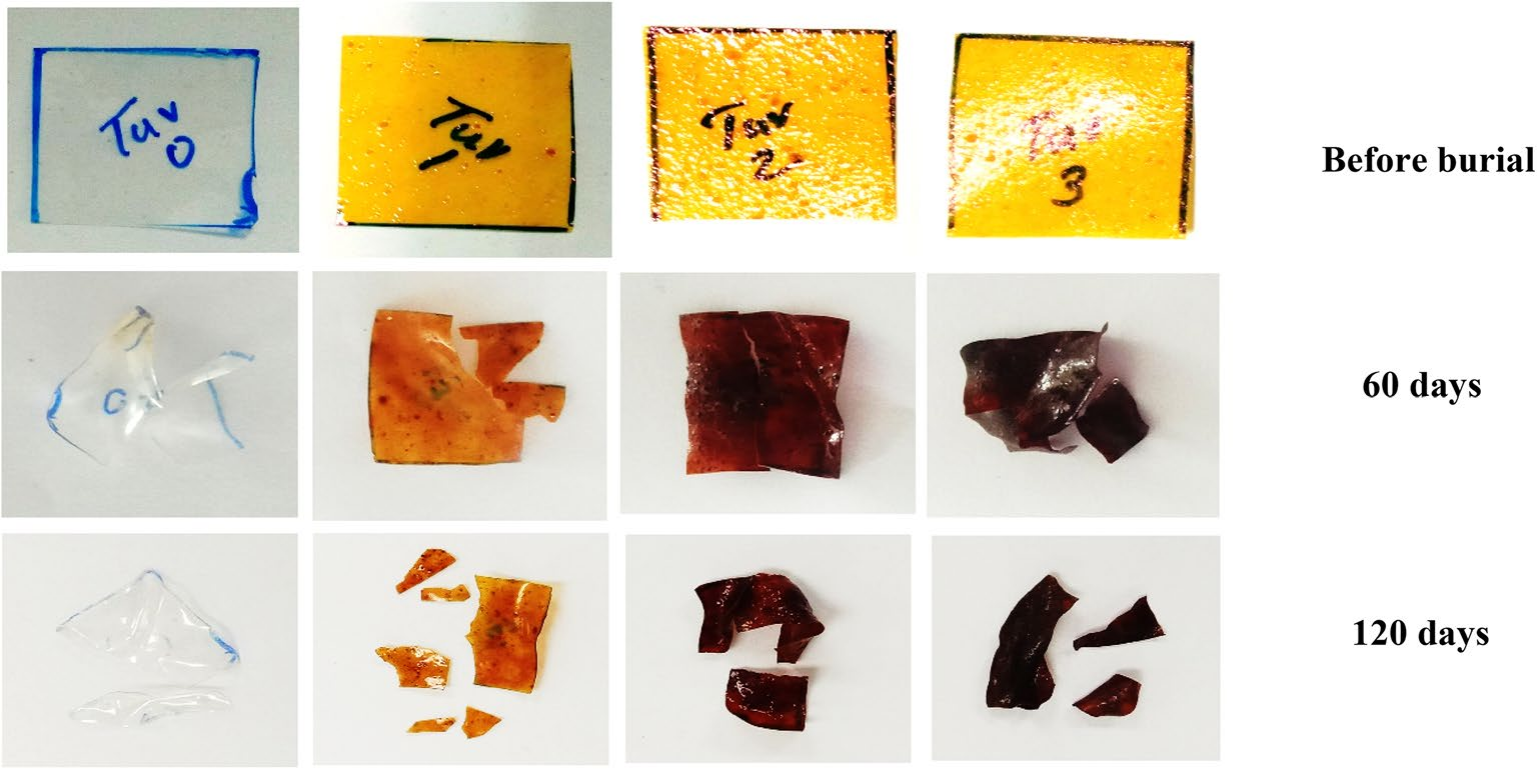
Images of soil biodegradability of PVA/CMC/TUR films at regular intervals.

We also compared the morphology of PVA/CMC film in the absence and presence of turmeric. A smooth and homogenous surface was obtained for neat PVA/CMC film. However, after loading with turmeric, the morphology of PVA/CMC film changed and particle with different size and shape is clearly visible in the SEM micrograph of PCT-1, PCT-2 and PCT-3 (Fig. 8). The particle distribution is found to increase with turmeric content. This could be taken as evidence for the successful incorporation of turmeric in the polymer matrix. Being rich in turmeric content, the PCT-3 films has rougher surface than PCT-2 and PCT-1 films^56^. These observations were complementary to optical microscopic analysis and confirmed the successful incorporation of turmeric in the film (Fig. 9). The degradation behaviour of the turmeric loaded films is shown in Fig. 10. We also accessed the biodegradability of the film by soil degradation analysis and the weight loss of PVA/CMC/TUR film is presented in Table 4. The neat PVA/CMC film have slow degradation rate. However, we can observe a significant improvement in the biodegradability after incorporating turmeric into PVA/CMC film. Turmeric induces biodegradability and therefore PVA/CMC/TUR film degrade relatively fastly and accelerate the weight loss. In fact, 32% weight loss was noted for film loaded with 3 wt% of turmeric. Similar observation was reported by D’souza et al. and Jaramillo et al.^51,64^. They observed that the addition of Yerba mat extract into the polymer matrix, increases the rate of soil degradation^64^.

**Table 4:** Weight loss of PVA/CMC/TUR films at different periods.

Samples	Weight loss (g)		
	1st day	60 days	120 days
PCT-0	0.06	0.048	0.028
PCT-1	0.09	0.073	0.049
PCT-2	010	0.074	0.052
PCT-3	0.08	0.052	0.025

**Figure 11:**
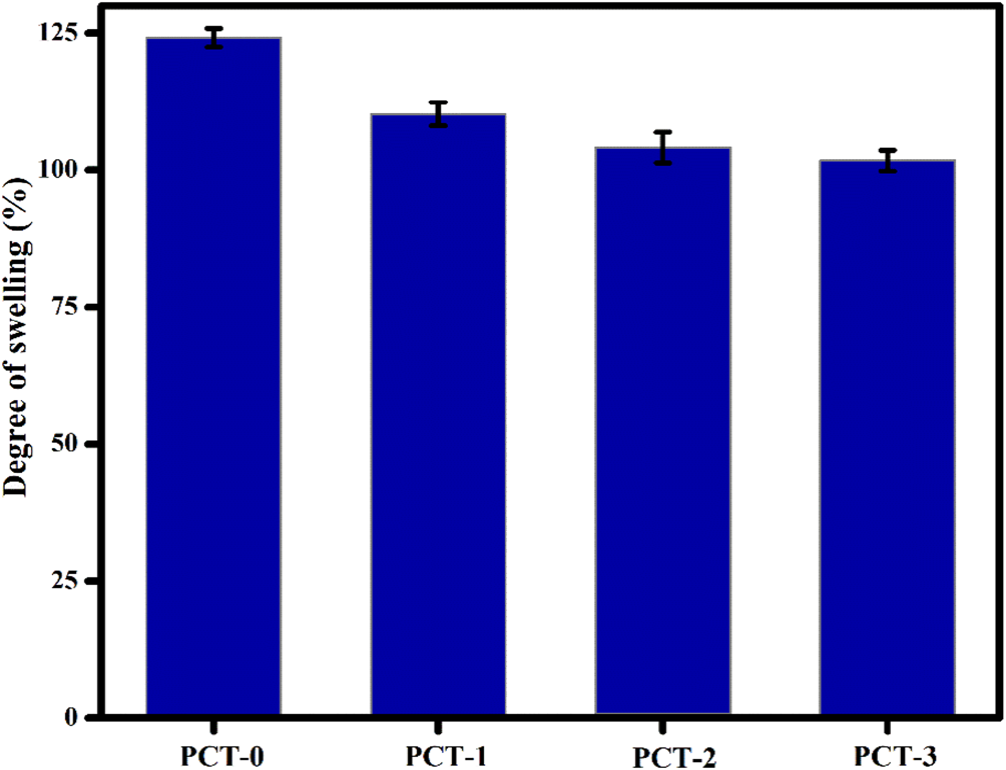
Degree of swelling of PVA/CMC/TUR films.

**Figure 12:**
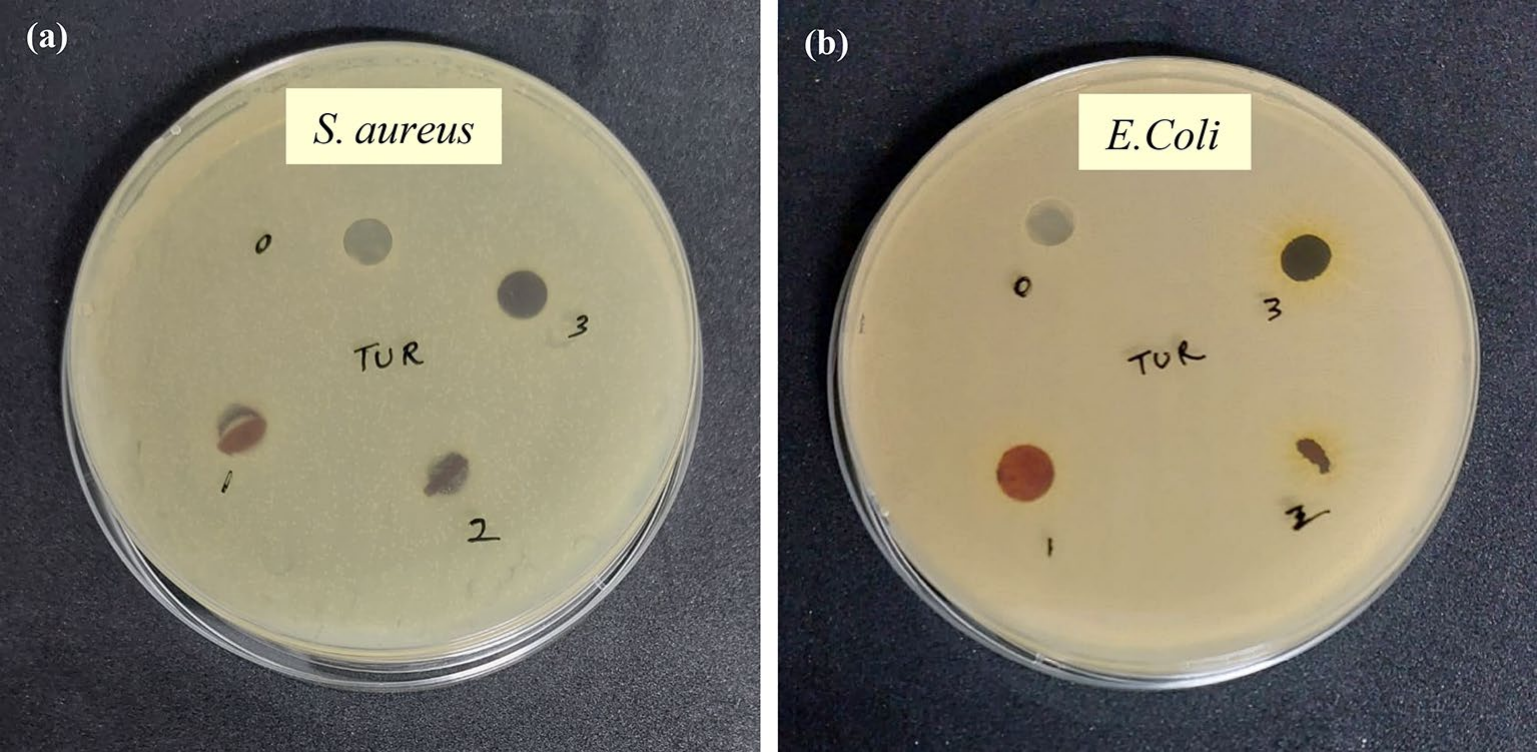
Photograph of antibacterial activity of zeolite loaded PVA/CMC/TUR films against (**a**) gram-positive (*S. aureus*) and (**b**) gram-negative (*E. coli*) bacteria: (0) PCT-0 (1) PCT-1 (2) PCT-2 (3) PCT-3.

The swelling behaviour of PVA/CMC/TUR film in water was studied and percentage of swelling is displayed in Fig. 11. Owing to the presence of hydroxyl groups, CMC and PVA have high affinity for water and consequently swell to a high rate. After cross linking, the water solubility and water affinity of PVA/CMC/TUR films declines. Thus, we observed a low swelling percentage for crosslinked PVA/CMC system. The addition of turmeric powder into PVA/CMC polymer matrix, further decreased its swelling capacity. The low degree of swelling value for turmeric loaded film as compared with neat PVA/CMC film is ascribed to the hydrophobic nature of turmeric^65^. The antibacterial activity of PVA/CMC/TUR films was investigated for *E.coli* and *S.aureus* bacteria and the photographs obtained by inhibition zone method is shown in Fig. 12. The neat PVA/CMC film did not show any inhibition zone against *E.coli* and *S.aureus* bacteria. However, the antibacterial activity has significantly increased with the incorporation of turmeric into PVA/CMC film. The enhancement in the inhibition zone is attributed to the presence of antibacterial agents namely curcuminoids and terpenoids in the turmeric. The films with good mechanical property are preferred for industrial applications, therefore we checked the mechanical property of the film and the effect of turmeric content on the tensile strength and elongation at break of the films was reported Table 5. With increase in turmeric content, a notable increase in the mechanical properties was observed. The turmeric intercalates between the polymer matrix and thereby enhance the tensile strength of the PVA film. It should also be noted the elongation at break values also increased with turmeric content from 1 to 3 wt%. This result could be related to the FTIR observation and confirm the chemical interaction between the polymer and the turmeric^55^.

**Table 5:** Mechanical properties of PVA/CMC/TUR films.

Samples	Tensile Strength (MPa)	Elongation at break (%)
PCT-0	10.55 ± 2.54	27.61 ± 2.42
PCT-1	15.36 ± 1.72	43.61 ± 2.23
PCT-2	19.96 ± 4.25	45.95 ± 5.38
PCT-3	38.4 ± 1.65	48.04 ± 1.87

### Adsorption studies

**Figure 13:**
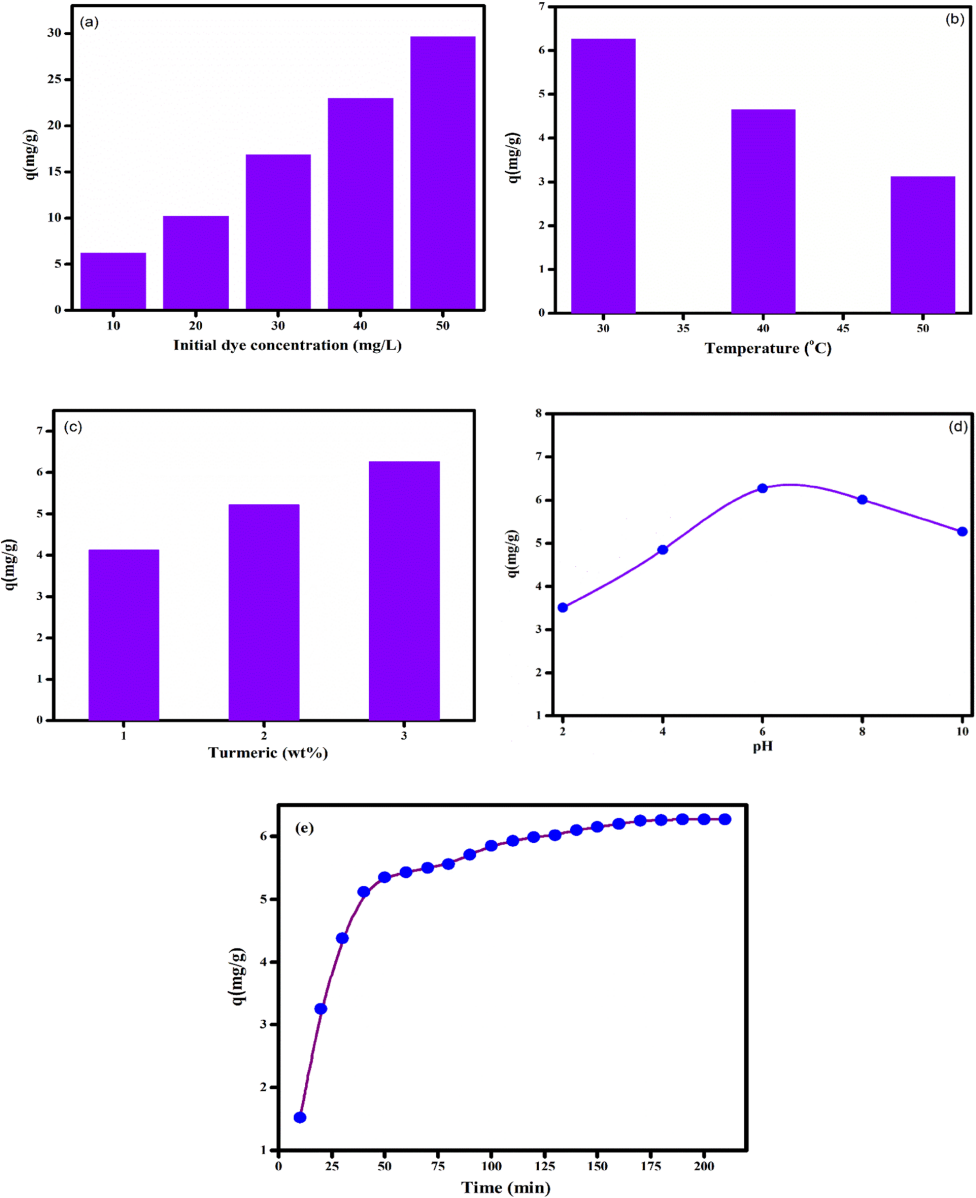
Effect of different parameters such as (**a**) initial dye concentration; (**b**) temperature; (**c**) turmeric dosage; (**d**) pH and (**e**) contact time on the MB removal dye on PVA/CMC/TUR films (Experimental condition: adsorbent dosage = 3 wt%, initial dye concentration = 10 mg/L, contact time = 170 min, pH = 6 and temperature = 30 °C).

**Figure 14:**
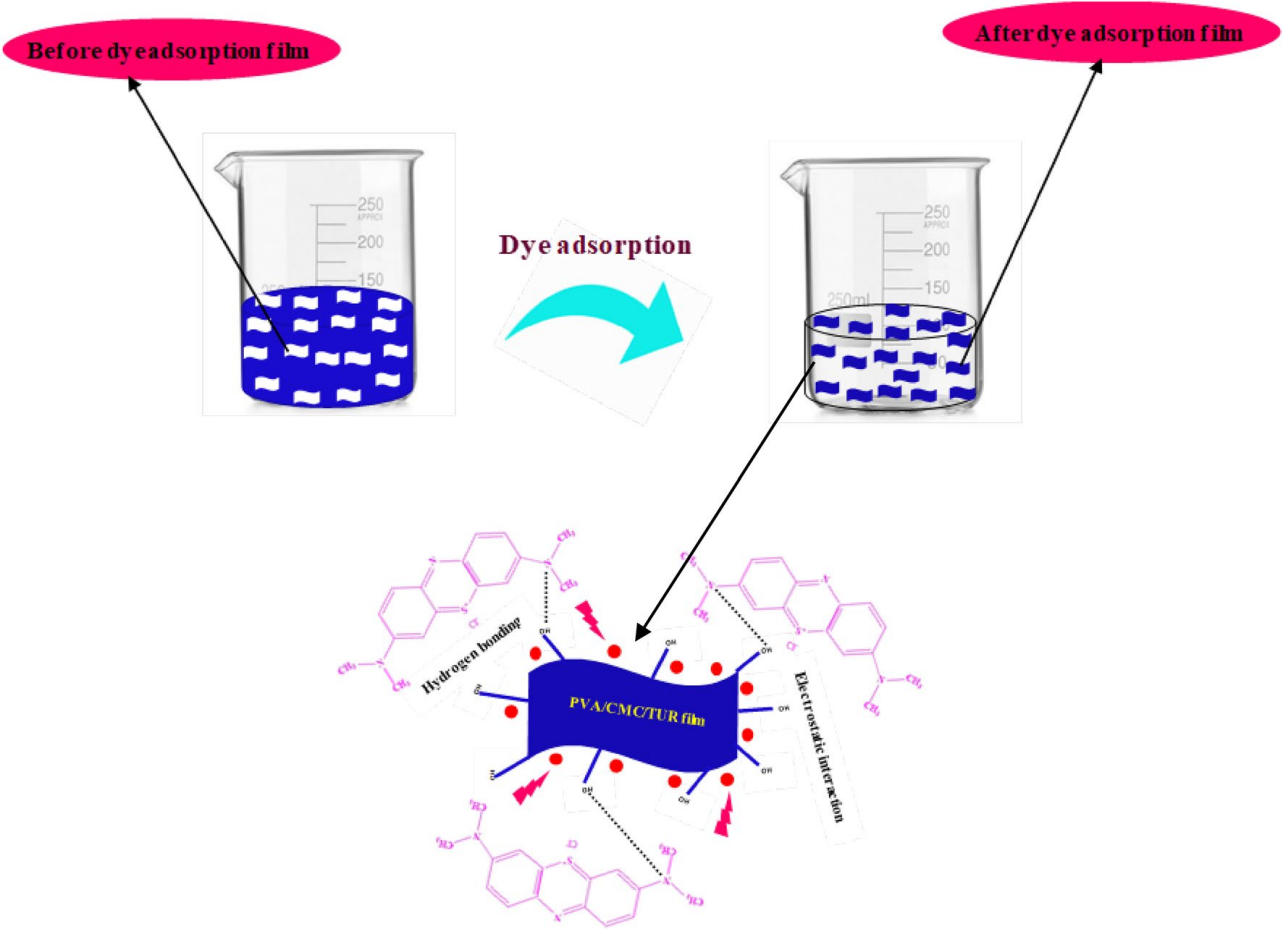
MB dye molecules interact with the PVA/CMC/TUR film.

The initial dye concentration is reported to have a major influence on the dye uptake ability of the film. Previous reports shows that it can either increase or decrease the dye removal efficiency of the adsorbent system. Apparently, an increase in initial dye concentration enhances the mass transfer of dye molecules and thereby increase the dye uptake capacity of the film^66^. On contrary, some reports suggest that the initial dye concentration have a negative influence on the removal percentage. In our case, we observed a significant increase in removal percentage with increase in MB concentration (Fig. 13a). In the light of the previous report^57^, we presume that at high initial concentration (50 mg L^-1^) there will be less resistance for the transfer of MB molecules from the solution to the surface of PVA/CMC/TUR film. Greater interaction between the dye molecules and the film leads to high removal percentage and high adsorption capacity^67^. Temperature is one of the crucial parameters for adsorption studies. Generally, high temperature favours adsorption process as it increases the diffusion of molecule from bulk to the surface. Increase in adsorption capacity with temperature also indicate the endothermic nature of adsorption. Some reports shows that elevated temperature has a detrimental effect on the interaction between the adsorbate and the adsorbent. Therefore, with increase in temperature the adsorbed molecule desorbed from the surface of the adsorbate and thereby result in low adsorption capacity^68^. The effect of temperature on the MB adsorption on the film is shows in Fig. 13b. It can be seen that with increase in temperature, the adsorption capacity decreases. The maximum adsorption capacity of 6.27 mg/g was noted at low temperature. A low adsorption capacity (3.12 mg/g) was noted for film exposed to 50 °C. This is ascribed to the fact that high thermal energy facilitates the desorption of MB molecule from the surface. The adsorbent dosage is another parameter which plays a crucial role in the adsorption process. The effect of adsorbent dosage on the dye removal efficiency was evaluated under fixed condition i.e., room temperature (30 °C), contact time (170 min) and initial dye concentration (10 mg L^-1^). It is clearly seen that with increase in the adsorbent dosage (1–3 wt%), the dye removal percentage increases (Fig. 13c). The maximum dye removal (83%) was achieved at 3wt% of adsorbent dosage. After 3 wt%, the adsorption capacity remains constant due to the saturation of adsorption site. The result thus shows that turmeric dosage plays a crucial role in the adsorption behaviour of the film^69^. Our result is similar to the reports of Kumari et al.^70^. Here, 93% of CV dye was removed at high adsorbent dosage (0.4 gm). In another study using zeolitic imidazolate framework (ZIF-8)/graphene oxide (GO)/carbon nanotubes (CNTs) hybrid nanocomposites, good removal efficiency was reported at high adsorbent dosage^71^. It is a well-known fact that pH value has considerable effect on the adsorption process. This prompted us to study the effect of pH on the dye adsorption process. We varied the pH of the medium from 2 to 10 and the adsorption capacity at different pH value was recorded (Fig. 13d). On varying the pH from acidic to basic range, we can observe an increase in the adsorption capacity. Acidic medium induces protonation of the film and thereby generates an unfavourable condition for adsorption, due to the electrostatic repulsion between the positively charged film and the cationic MB molecules. On the other hand, in basic medium, the surface gets deprotonated and thereby facilitate the adsorption of cationic dyes. Dai et al. reported maximum CR adsorption capacity (12,000 mg/g) at basic medium for calcium rich biochar^72,73^. However, at very high pH, there will be competition between the adsorbent and hydroxyl ion (OH–) ions for the cationic dye. Consequently, the dye adsorption tends to decline at high pH. In the current study, we obtained maximum dye adsorption at pH-6. The schematic representation of the interaction between the dye molecule and the film is shown in Fig. 14. The effect of contact time on the dye adsorption was studied and the result is displayed in Fig. 13e. In the initial contact time, the number of vacant sites present on the adsorbent surface will be more and therefore the MB molecules can easily be adsorbed on the PVA/CMC/TUR film. Hence, a rapid uptake of MB dye onto the films was noted in the initial 30 min. With the passage of contact time, the dye adsorption rate slows down and the adsorption reached saturation within 170 min of exposure time^74^.

### Adsorption kinetics

The rate of dye adsorption of MB on the films was accessed using three models pseudo-first order (PFO) and pseudo-second order (PSO) and intraparticle diffusion model (IPD). The linear form of the kinetic model is provided in the Table 6. The adsorption parameters like K_1_, K_2_, K_id_, C and q_e_ where calculate from the slope and intercept of the corresponding plots of the kinetic models.

**Table 6:** Differential and linear forms of pseudo-first order (PFO), pseudo-second order (PSO) and intraparticle diffusion models (IPD).

Kinetic models	Linear equation	Plot
PFO	$\mathrm{log}\left({\mathrm{q}}_{\mathrm{e}}-{\mathrm{q}}_{\mathrm{t}}\right)={\mathrm{logq}}_{\mathrm{e}}-\frac{{\mathrm{K}}_{1}\mathrm{t}}{2.303}$	log (q_e_-q_t_) versus time
PSO	$\frac{\mathrm{t}}{{\mathrm{q}}_{\mathrm{e}}}=\frac{1}{{\mathrm{K}}_{2}{\mathrm{q}}_{\mathrm{e}}^{2}}+\frac{\mathrm{t}}{{\mathrm{q}}_{\mathrm{e}}}$	t/qt versus time
IPD	${\mathrm{q}}_{\mathrm{t}}={\mathrm{K}}_{\mathrm{id}}{\mathrm{t}}_{ }^{1/2}$+ C	q_t_ versus t^1/2^

k_1_ (min^-1^), is the pseudo-first order rate constant, k_2_ (min^-1^), is the pseudo-second order rate constant, K_id_ is the intraparticle diffusion rate constant, q_t_ (mg g^-1^) and q_e_ (mg g^-1^) are the amounts of dye adsorbed at time t (min) and equilibrium, respectively.

**Figure 15:**
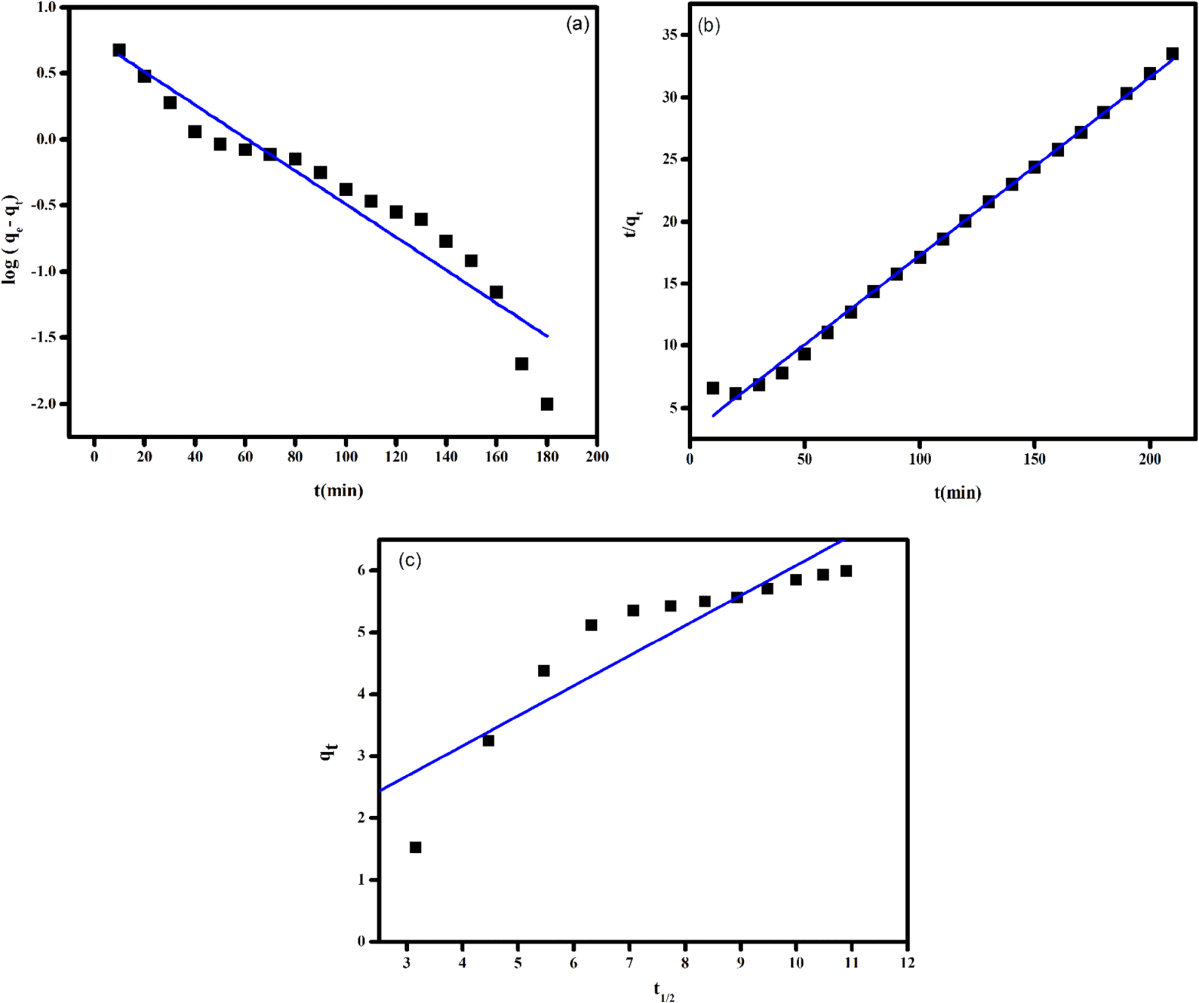
Linear fitting plots of (**a**) pseudo-first order kinetic (**b**) pseudo-second order kinetic and (**c**) intraparticle diffusion models of MB dye for the PVA/CMC/TUR films.

The PFO model corresponds to physisorption, whereas PSO model propose chemisorption. The IPD is mainly used for porous adsorbent system. The rate controlling step in IPD model is internal diffusion of adsorbate from the film to the pores of the adsorbent system. The adsorption is said to follow IPD, if the linear plot of q_t_ vs t^1/2^ passes through the origin i.e., when intercept is zero. The experimental data was fitted into these models and the obtained kinetic parameters is tabulated in Table 7. It is appreciated from Fig. 15 and Table 7 that the experimental data fitted well with PSO model with high correlation coefficient (0.997). The adsorption capacity obtained from the PSO model also matches well with the experimental value.

**Table 7:** Adsorption kinetic parameters of MB on PVA/CMC/TUR film.

Exp	Pseudo-first order			Pseudo-second order			Intra-particle diffusion		
q_e_, exp (mg g^-1^)	K_1_ (min^-1^)	q_e_ (mg g^-1^)	R^2^	K_2_ (g mg^-1^ min^-1^)	q_e_ (mg g^-1^)	R^2^	K_id_ (g mg^-1^ min^-1/2^)	C (mg g^-1^)	R^2^
6.27	− 0.287	5.77	0.955	0.0069	6.98	0.997	0.48	1.21	0.90

### Adsorption isotherm

In the present study, Langmuir, Freundlich, Temkin and Dubinin-Radushkevich (D-R) isotherm models was employed for studying the adsorption mechanism. The linear and non-linear equation of the model along with its parameter is displayed in Table 8.

**Table 8:** Linear and non-linear models of Langmuir, Freundlich, Temkin and D-R isotherms.

Models	Linear	Non-linear equation	Plot
Freundlich	*In* $\left({q}_{e}\right)={In K}_{F}+\frac{1}{n}In \left({C}_{e}\right)$	${q}_{e}={K}_{F}{C}_{eq}^{1/n}$	*In* ${q}_{e}vs In {C}_{e}$
Langmuir	$\frac{{C}_{e}}{{q}_{e}}=\left(\frac{{C}_{e}}{{q}_{m}}\right)+\left(\frac{1}{{K}_{L}*{q}_{m}}\right)$	${q}_{e}=\frac{{q}_{m}{K}_{L}{C}_{e}}{1+{K}_{L}{C}_{e}}$	$\frac{{C}_{e}}{{q}_{e}}vs{C}_{e}$
Temkin	*B* = *B*_*T*_*InA*_*T*_ + *B*_*T*_*InC*_*e*_	*q*_*e*_ = *B*_*T*_*.In (K*_*T*_*.C*_*e*_*)*	*q*_*e*_* vs ln C*_*e*_
D-R	*In* ${q}_{e= }{Inq}_{m}- \beta {\varepsilon }^{2}$	${q}_{e}$= ${q}_{m}$ *exp (-β *${\varepsilon }^{2}$*)*	*ε*^*2*^* vs In* ${q}_{e}$

C_e_ is the equilibrium concentration of adsorbate (mg/l), q_e_ is the equilibrium adsorption capacity of the adsorbent (mg/g) and q_m_ is the maximum adsorption capacity (mg/g). K_L_ and K_F_ are Langmuir and Freundlich constants (L/mg). B and K_T_ is the Temkin isotherm constant. β is the D-R constant (mol^2^J^-2^) which is associated with average adsorption energy per mole of the adsorbate, q_m_ is the maximum adsorption capacity (mg/g), ε is the Polanyi potential (kJ mol^− 1^).

**Figure 16:**
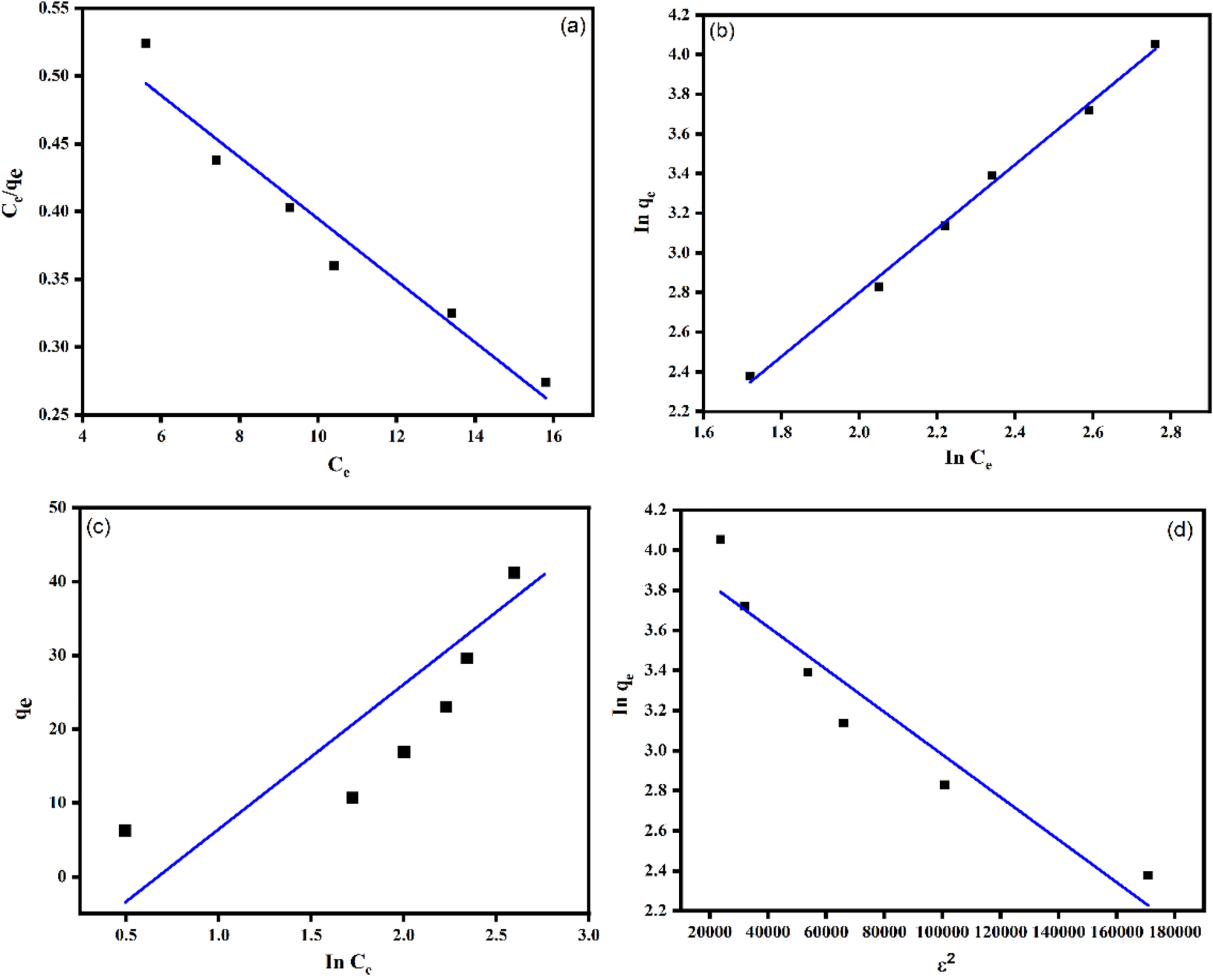
Linear isotherm plots of (**a**) Langmuir; (**b**) Freundlich; (**c**) Temkin and (**d**) D-R isotherm models for the MB dye adsorption onto PVA/CMC/TUR films.

Langmuir model assumes a homogenous adsorption surface with identical adsorption site. The value of q_m_ and K_L_ are determined from the slope and intercept of the linear plot C_e_/q_e_ vs C_e_ (Fig. 16a). This model further assume that the adsorbed molecules have no interaction with each other and therefore describe a chemisorption process.

Freundlich isotherm on the other hand emphasize the heterogeneity of the adsorption surface. Therefore, it is most suitable to describe multilayer adsorption. The important parameter related to Freundlich isotherm are K_F_ and n, which is obtained from the linear plot of ln q_e_ against ln C_e_ (Fig. 16b). K_F_ is related to the maximum adsorption capacity (mg/g) and ‘n’ gives the adsorption intensity.

Temkin isotherm model is another important isotherm model which suggest indirect interaction between the adsorbed molecules. In this model, the heat of adsorption of all adsorbed molecules would decreases linearly with increase in the initial coverage of the adsorbate. The parameter obtained from Temkin models namely isotherm constant B and A_T_ is very essential to understand the adsorption behaviour (Fig. 16c). The isotherm constant is directly related to heat of adsorption whereas the A_T_ (L g^-1^) value indicate the maximum binding energy. DR isotherm model is another important isotherm model which is derived from microporous filling theory. This model is generally applied for solid and liquid heterogenous adsorbent system (Fig. 16d). The linear form of DR isotherm is given in Table 8.

**Figure 17:**
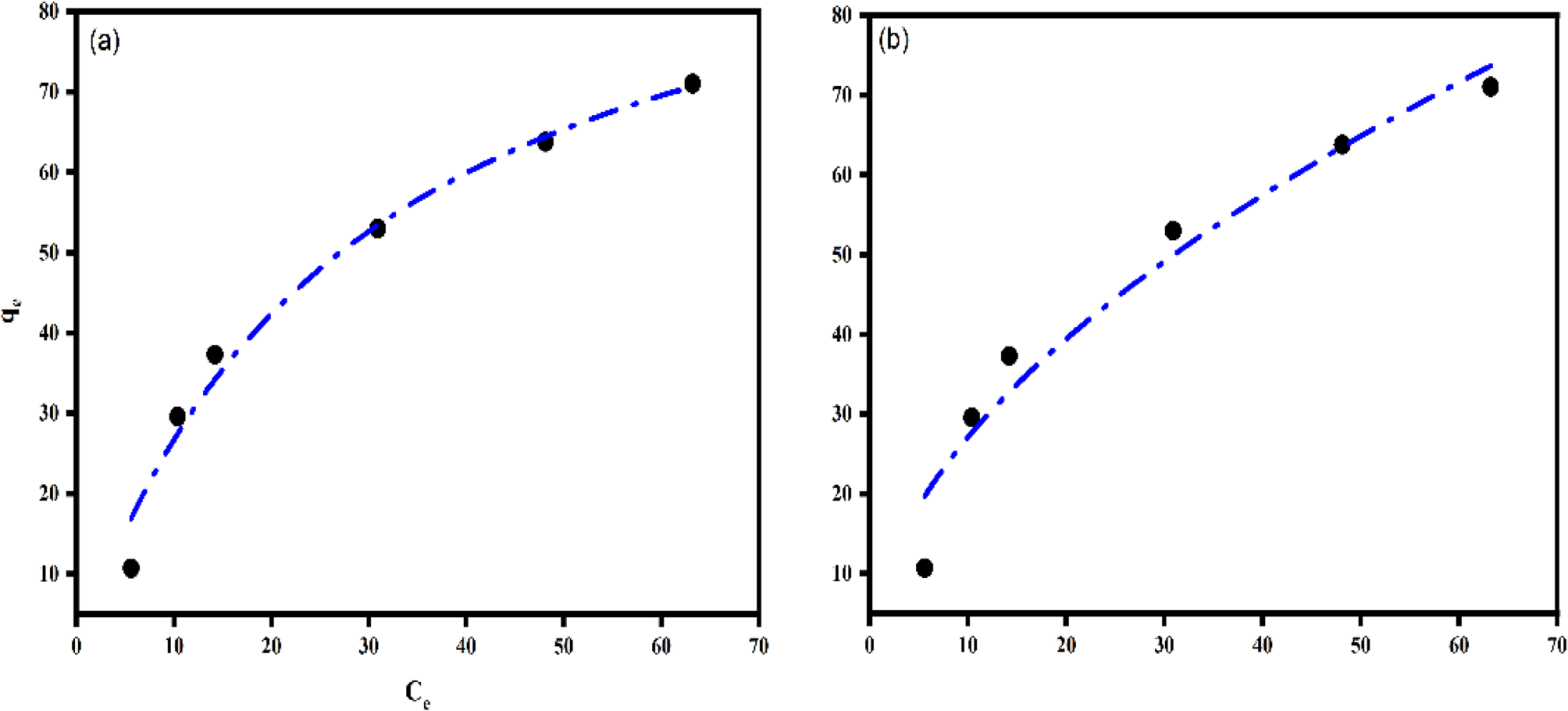
Non-linear isotherm plots of (**a**) Langmuir and (**b**) Freundlich isotherm models for the MB dye adsorption onto PVA/CMC/TUR films.

The experimental adsorption data of PVA/CMC/TUR film was fitted on the aforementioned isotherm models. The experimental data fitted well in both Freundlich (R^2^ = 0.991 and 0.972 for linear and non-linear form respectively) and Langmuir (R^2^ = 0.97 and 0.97 for linear and non-linear form respectively) models (Tables 9 and 10) (Fig. 17). The least fit of the experimental data in Temkin and D-R model suggests that this model is not a valid model to explain the adsorption of MB onto the film.

**Table 9:** Langmuir, Freundlich, Temkin and D-R linear isotherm parameters on PVA/CMC/TUR films.

Experimental	Langmuir			Freundlich			Temkin			D-R		
	q_m_(mgg^-1^)	K_L_	R^2^	K_F_ (mgg^-1^)	n	R^2^	A_T_ (L g^-1^)	B	R^2^	q_DR_	β	R^2^
6.27	44	0.036	0.97	1.54	0.62	0.99	1.95	19.6	0.82	56.8	1.06	0.95

**Table 10:** Langmuir and Freundlich non-linear isotherm parameters on PVA/CMC/TUR films.

Experimental	Langmuir			Freundlich		
	q_m_(mgg^-1^)	K_L_	R^2^	K_F_ (mgg^-1^)	n	R^2^
6.27	102	0.0353	0.97	7.74	1.84	0.97

### Thermodynamics studies

A proper knowledge of thermodynamic parameter is required to design an adsorbent system. The thermodynamics of MB adsorption on the film was studied at different temperature and the thermodynamics parameters were calculated. The Gibbs free energy (ΔG), enthalpy (ΔH) and entropy (ΔS) of adsorption were calculated using the following Eqs. (4) and (5).

4 $$\mathrm{ln}\frac{{\mathrm{q}}_{\mathrm{e}}}{{\mathrm{C}}_{\mathrm{e}}}= -\frac{\Delta \mathrm{H}}{\mathrm{RT }}+ \frac{\Delta \mathrm{S}}{\mathrm{R}}$$

The Gibbs free energy (ΔG) can be evaluated using the equation:

5 $$\Delta \mathrm{G}= \Delta \mathrm{H}-\mathrm{T}\Delta \mathrm{S}$$

where T is absolute temperature in Kelvin (K), R is the universal gas constant (8.314 J/ mol/ K). ΔG is the free energy of sorption (kJ/mol), ΔS(J/mol) and ΔH(kJ/mol) are respectively the entropy and enthalpy of sorption.

The free energy data is used to understand the feasibility or spontaneity of adsorption process. The negative free energy represents a spontaneous adsorption process. The enthalpy is related to the energy adsorbed or released during the adsorption process. A negative enthalpy indicates exothermic nature whereas positive value indicates endothermic nature of adsorption. The entropy of the system represents the randomness or disorder. A positive entropy can be taken as strong evidence of disorderness in the system. The thermodynamic parameter obtained for MB adsorption is illustrated in Table 11. The negative value of free energy suggests feasibility of MB adsorption on the PVA/CMC/TUR film. The negative enthalpy demonstrates an exothermic adsorption, meanwhile positive entropy indicates randomness at solid/liquid interface.

**Table 11:** Thermodynamics parameters for MB adsorption on PVA/CMC/TUR film.

ΔG(kj/mol)			ΔH(kj/mol)	ΔS(j/mol)
303 K	313 K	323 K	131.19	− 404
− 253	− 257.6	− 261.6		

### Reusability

**Figure 18:**
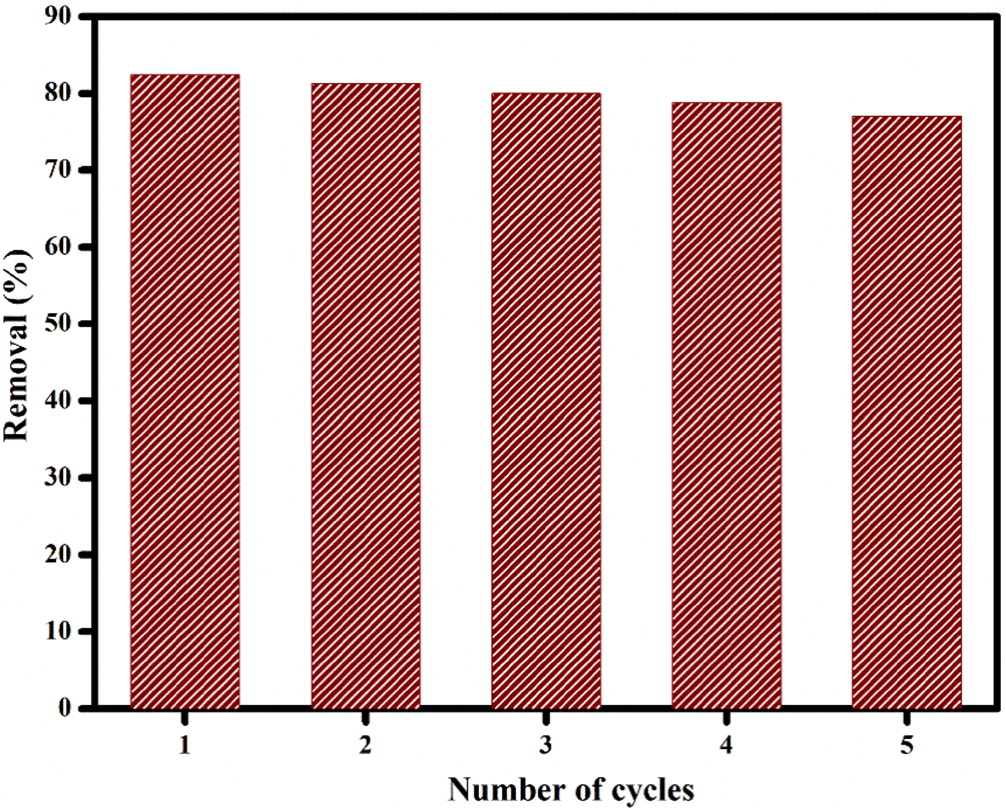
Reusability test of MB dye on PVA/CMC/TUR film.

For industrial purposes, the reusability of the adsorbent is an important criterion. Figure 18 show the reusability property of PVA/CMC/TUR film with number of cycles. The reusability of PVA/CMC/TUR film was checked using 0.1 N HCl as desorbing agent. The film retained good adsorption efficiency (77%) even after 5 five adsorption–desorption cycles. The minor reduction noted after each cycle could be due to the incomplete desorption of dye from the surface of the film (Fig. 18).

### Comparison with other adsorbents

We also compared the adsorption capacity of PVA/CMC/TUR film with other conventional adsorbent. As it is reported in Table 12, PVA/CMC/TUR film exhibit high adsorption property than other adsorbents. Therefore, PVA/CMC/TUR adsorbent could be used as cost-effective, eco-friendly material for wastewater treatment.

**Table 12:** Comparison studies of previous work on MB dye on various adsorbents.

Adsorbents	q_e_ (mg/g)	References
Caulerpa racemosa var.cylindracea	5.23	^75^
Coal fly ash	2.88	^76^
Graphite oxide	0.74	^77^
Cotton alk	0.024	^78^
Coir pith carbon	5.87	^79^
PVA/CMC/TUR	6.27	This work

## Conclusion

In summary, a novel eco-friendly bio adsorbent was developed from PVA, CMC and turmeric for the adsorption of MB from aqueous medium. The film was successfully developed through solvent casting technique. The physical and chemical properties of the film was reported. The PVA/CMC/TUR films are stable and possess good thermal stability and biodegradability. The incorporation of turmeric powder into PVA/CMC polymer matrix enhanced the adsorption capacity of the film. The adsorption capacity of the film was optimized at different parameters such as contact time, pH, temperature, initial dye concentration and turmeric dosage. Maximum dye adsorption (83%) was achieved at initial dye concentration of 10 mg/L with contact time 170 min at room temperature. The adsorption isotherm studies revealed that both Langmuir and Freundlich isotherm is suitable for the MB adsorption. The kinetic follows pseudo-second order model. Thermodynamics studies demonstrate the spontaneous nature of adsorption. The film possesses good reusability property up to 5 cycles and therefore it is a potential candidate for dye removal from industrial waste streams.

## Data Availability

All data generated or analysed during this study are included in this manuscript.
